# Epidemic Ophthalmia, with Papers upon Allied Subjects

**Published:** 1896-12

**Authors:** 


**Epidemic Ophthalmia, with Papers upon Allied Subjects.** By SYDNEY STEPHENSON, M.B. Pp. viii., 278. Edinburgh: Young J. Pentland. 1895.

The symptoms, diagnosis, and management of epidemic ophthalmia, as it invades schools, workhouses, barracks, &c., are here discussed with great detail. Dr. Stephenson deals with: (1) the various affections grouped under this name and how to distinguish between them; (2) the ways in which those affections are spread; and (3) the measures that have been found most successful in dealing with these several conditions. The author's knowledge of this subject is based upon his large experience at the Ophthalmic School, Hanwell, and his well-known interest in it has for some time given prominence to his views, whether written or in debate. The book is equally valuable to ophthalmic specialists, to school doctors, or to those concerned in the management of institutions in which large numbers of individuals are housed together, particularly under conditions of overcrowding or imperfect sanitation.

